# HIF2α-dependent Dock4/Rac1-signaling regulates formation of adherens junctions and cell polarity in normoxia

**DOI:** 10.1038/s41598-024-62955-7

**Published:** 2024-05-27

**Authors:** I. Raykhel, V.-P. Ronkainen, J. Myllyharju, A. Manninen

**Affiliations:** 1 Disease Networks Research Unit, Faculty of Biochemistry and Molecular Medicine, Biocenter Oulu, University of Oulu, Oulu, Finland; 2 Extracellular Matrix and Hypoxia Research Unit, Faculty of Biochemistry and Molecular Medicine, Biocenter Oulu, University of Oulu, Oulu, Finland

**Keywords:** Hypoxia, HIF, Kidney epithelial polarity, Adherens junction, Cancer, Adherens junctions, Apicobasal polarity, RHO signalling, Stress signalling

## Abstract

Hypoxia-inducible factors (HIF) 1 and 2 regulate similar but distinct sets of target genes. Although HIFs are best known for their roles in mediating the hypoxia response accumulating evidence suggests that under certain conditions HIFs, particularly HIF2, may function also under normoxic conditions. Here we report that HIF2α functions under normoxic conditions in kidney epithelial cells to regulate formation of adherens junctions. HIF2α expression was required to induce Dock4/Rac1/Pak1-signaling mediating stability and compaction of E-cadherin at nascent adherens junctions. Impaired adherens junction formation in HIF2α- or Dock4-deficient cells led to aberrant cyst morphogenesis in 3D kidney epithelial cell cultures. Taken together, we show that HIF2α functions in normoxia to regulate epithelial morphogenesis.

## Introduction

Hypoxia-inducible factors (HIFs) are transcription factors that predominantly regulate the adaptation to hypoxia at the cellular or organismal level. HIFs consist of two subunits, termed HIFα and β. In hypoxic conditions HIFα and HIFβ form a dimer that translocates to the nucleus and binds to hypoxia-response elements (HRE) inducing the transcription of more than hundred target genes involved in erythropoiesis, angiogenesis, anaerobic metabolism, cell differentiation and survival^1–4^. The HIFα family includes three homologues—HIF1α, HIF2α and HIF3α. HIF2α, also named endothelial PAS domain protein-1 (EPAS1), was discovered in 1997 and shares 48% sequence identity with HIF1α^5^. Among HIF target genes there are exclusive HIF1α targets, exclusive HIF2α targets, and genes that are responsive to both HIF1α and HIF2α^6–9^. Recent studies revealed that HIFs can interact via different (non-HRE-mediated) mechanisms^1^. HIF1α and HIF2α can play non-overlapping and even opposite biological roles due to their unique target genes and different requirement of oxygen for activation^6^. HIF1α requires acute hypoxic conditions for stabilization, in contrast, HIF2α may accumulate under modest hypoxia or even normal physiological oxygen levels^6^. HIF1α is widely expressed while HIF2α is preferably expressed in specific cell types such as endothelial cells and hypoxic kidney epithelium^5,10–13^. The expression of HIF2α is commonly increased in various human tumors as well as certain cancer stem cell populations^14–16^.

Despite the established role in hypoxia response, HIF2α is also considered to function under normoxic conditions. In cancer stem cells, HIF2α activates downstream genes without hypoxia stimulation in vitro and in vivo^16^. It was reported that HIF2α is expressed in gastric cancer cells under normoxic conditions^17^. Moreover, neuroblastoma cells express high levels of HIF2α regulating proliferation and tumor growth^18^. HIF2α has been suggested to participate to the maintenance of stem cell pluripotency, presumably via regulation of epithelial-to-mesenchymal transition (EMT)^14,19^. Interestingly, HIF2α was reported to contribute to adherens junction (AJ) integrity in the lung by regulating the expression of vascular endothelial protein tyrosine phosphatase (VE-PTP)^20^. VE-PTP activity reduced VE-cadherin endocytosis thereby augmenting endothelial AJ integrity. Endothelium in the different tissues delivers oxygen to epithelial organs with vastly different partial pressure of oxygen. Much of the research has focused on the role of HIFs in endothelial cells but less is known about how HIFs function in the epithelial cells. Loss of epithelial polarity, including perturbed AJ integrity, within the tumor tissue is known to correlate with aggressiveness of the tumor cells^21^. Previous studies have reported that VHL-loss driven HIF stabilization in renal cancer led to downregulation of E-cadherin, a hallmark of EMT^22^. It was proposed that HIF activation in VHL-deficient cancer cells, with both HIF1α and HIF2α playing a role, promoted tumorigenesis by depleting E-cadherin from maturing AJs. Whether HIF2α is involved in the regulation of E-cadherin levels and epithelial cell polarity in VHL-expressing kidney epithelium in normoxic conditions remains incompletely understood.

Here, we have studied the potential role of HIF2α in the regulation of epithelial polarity using a well characterized three-dimensional (3D) Madin–Darby canine kidney (MDCK) epithelial cell culture system^23^. We show that HIF2α levels in normoxic conditions upregulate the mRNA and protein expression levels of a Rac1 guanine nucleotide exchange factor (GEF) Dedicator Of CytoKinesis 4 (Dock4). Dock4-mediated activation of Rac1 is needed to support the formation of polarized MDCK cysts. Depletion of HIF2α or Dock4 in normoxia interfered with MDCK cystogenesis leading to formation of multilumen cysts. This study identifies Dock4 as a novel mediator of HIF2α-driven activation of Rac1 signaling and shows that HIF2α expression in normoxia maintains basal Rac1 activity necessary for proper AJ formation and cystogenesis of kidney epithelial cells.

## Results

### Depletion of HIF2α expression disturbs MDCK cyst morphogenesis in normoxia

Hypoxia response pathway is a critical driver of renal cancers and disruption of epithelial morphogenesis and/or polarity correlates with cancer progression. In our previous studies we have used an established epithelial polarity model, Madin Darby Canine Kidney (MDCK) cells to investigate the effects of hypoxia and HIF1α on epithelial polarity and morphogenesis in the kidney^24^. Here we use the same MDCK cyst model to study the role of HIF2α in epithelial polarity. For this purpose we generated three independent HIF2α knockdown (KD) MDCK cell lines by using retrovirus-mediated RNA interference (RNAi) methodology^25,26^. The knockdown efficiency, ranging from 60,7% to 93,4%, of the target HIF2α (*EPAS1/HIF2A* gene) mRNA, was confirmed by real-time quantitative PCR (RT-qPCR) (Table S1, Fig. S1a). MDCK cells infected with a retrovirus containing an empty short hairpin RNA (shRNA) expression cassette were used as a control in all experiments.

To examine the effect of HIF2α knockdown on the function of this protein we performed a RT-qPCR analysis of selected known HIF target genes involved in hypoxia response. Whereas the control cells responded to hypoxia (1% O_2_, 48 h) by upregulating of the mRNA levels of vascular endothelial growth factor A (*VEGFA*), adrenomedullin (*ADM*), plasminogen activator inhibitor-1 (*PAI1*, also called *SERPINE1*) and lysyl oxidase (*LOX*), HIF2α-KD cells with the highest KD-efficiency (HIF2αKD#1) showed significantly reduced capacity to induce *VEGFA*, *ADM*, *PAI1* and *LOX* expression upon hypoxia (Fig. S1b). Analysis using HIF2αKO#2 and #3 cells displayed a similar trend although the inhibitory effects were not statistically significant for all the targets (data not shown).

Next we studied the effect of HIF2α-KD on epithelial morphogenesis by using 3D MDCK organotypic culture with basement membrane extract (BME) gels where control cells form highly polarized cysts in normal oxygen levels but display impaired cystogenesis leading multilumen phenotype under hypoxic conditions^24,27^. All cultures were grown in normoxic conditions for 1 h, after which half of the cultures were transferred into hypoxic conditions (1% O_2_) while the other half remained under normoxia. After 6 days in culture, cysts were fixed and stained for confocal microscopy analysis. The apical surface was visualized using antibodies targeting podocalyxin, a well-characterized apical membrane protein^28^. Actin cytoskeleton and nuclei were stained using TRITC-Phalloidin and DAPI, respectively. In normoxia, 78% of control cells were able to form hollow spherical cysts with a single apical lumen while only one third of the cysts formed a single lumen under hypoxia (Fig. S1c,d).

Surprisingly, only about one third of HIF2αKD#1 and ~ 60% of HIF2αKD#3 MDCK cells formed normal cysts under normoxic conditions and 95% of them were abnormal in hypoxia (Fig. S1c,d). This data suggests that HIF2α has a strikingly different function than reported for HIF1α. HIF1α upregulation in hypoxia was documented to activate the TGFβ-pathway via upregulation of BAMBI eventually leading to loss of epithelial polarity and HIF1αKD rescued normal cyst formation in hypoxic conditions^24^. In hypoxic conditions most of the MDCK WT and especially the HIF2αKD MDCK cysts lacked a central organized lumen (Fig. S1c,d). This finding may be due to the function of HIF1α mentioned above. Although HIF2αKD affected expression of hypoxia response genes (Fig S1b), our data suggests that HIF2α function is different from that of HIF1α as HIF2α appears to participate in the regulation of cyst morphogenesis also in normoxia.

**Figure 1:**
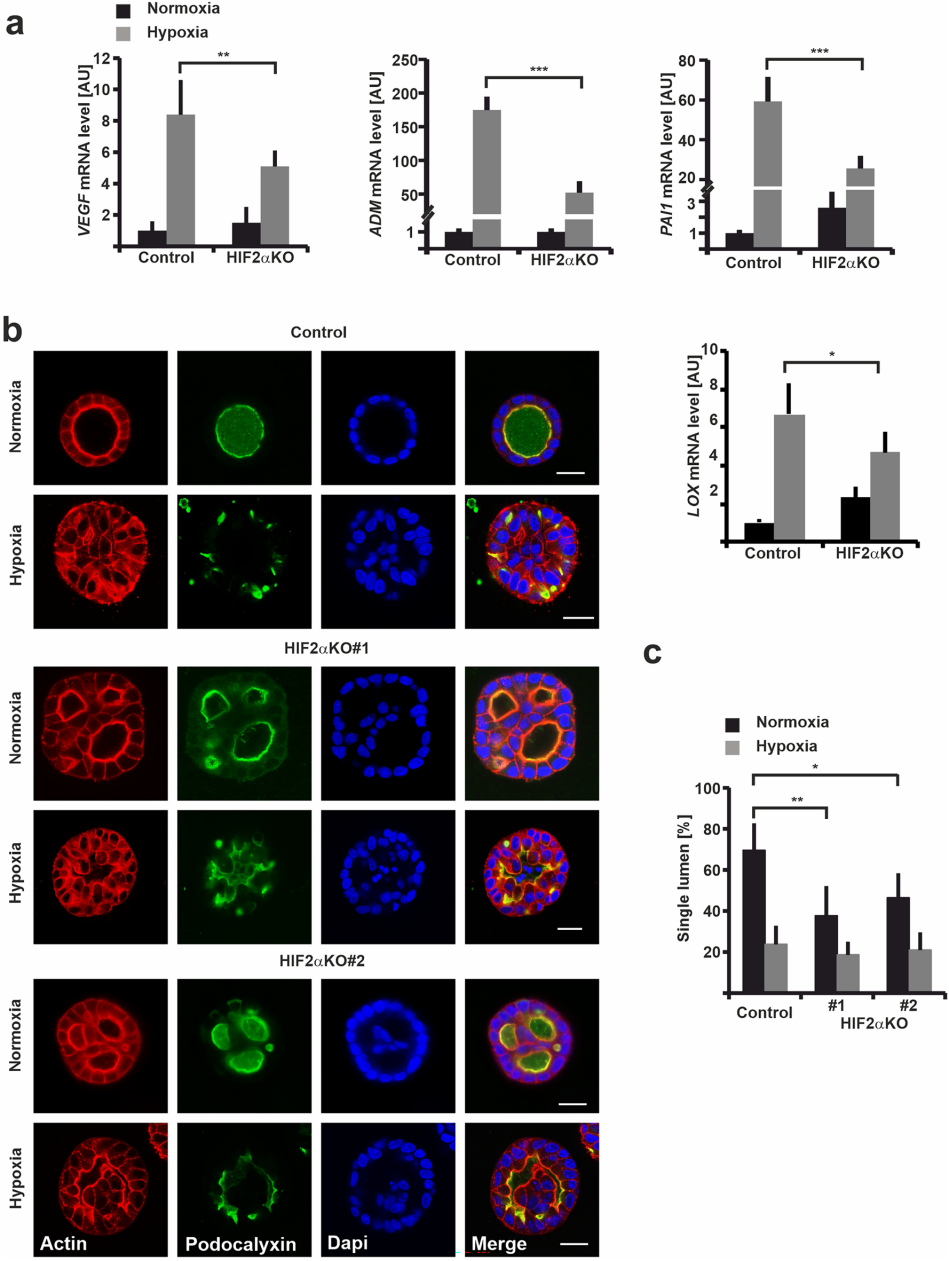
Knockout of HIF2A disrupts morphogenesis of MDCK cysts in normoxic conditions. (**a**) RT-qPCR analysis of the mRNA expression level of selected HIF target genes in 2D cultures of control and HIF2α-KO cell lines (#1 and #2 combined) under normoxic (N) and hypoxic (H, 48 h 1% O2) conditions. Data are presented as mean ± SD, n ≥ 3. **P < 0.001; *** P < 0.0001 (two-way ANOVA). (**b**) Control and HIF2α-KO MDCK cysts were grown in 3D under normoxia or hypoxia (48 h 1% O2) conditions. At day 6 the cysts were fixed and stained for an apical membrane protein podocalyxin (green), actin cytoskeleton (red) and nuclei (DAPI, blue). A single confocal slice from the middle of the cysts is shown. Scale bars: 20 µm. (**c**) Quantitation of the cyst phenotypes in control and HIF2α-KO MDCK cell lines grown as in (**b**). The percentage of cysts with single lumen was calculated and averaged from 4 to 8 independent experiments. A minimum of 160 cysts per sample was scored in each experiment. Data are presented as mean ± SD, n ≥ 4. *P < 0.05; **P < 0.001 (Fischer’s exact test).

Because the efficiency of HIF2α-depletion correlated with the penetrance of the phenotypes and due to the lack of well-functioning HIF2α antibodies to accurately monitor remaining protein levels, we wanted to confirm the HIF2α-KD data by designing two independent HIF2α-targeting gRNAs (Table S2) to generate HIF2α-knockout (KO) MDCK cell lines using CRISPR/Cas9 gene editing protocol^29^. Successful editing of the fourth exon on both HIF2α alleles leading to premature stop codon was confirmed by sequencing (Table S3) ensuring the loss of HIF2α expression in the cell clones selected to be used as biological replicates for further studies. MDCK cells transfected with a vector containing empty gRNA expression cassette were used as a control in all experiments. To examine the hypoxia response in control and HIF2α-KO cells we performed a RT-qPCR analysis of known HIF target genes. Whereas the control cells responded to hypoxia (1% O_2_, 48 h) by upregulation of the mRNA levels of *VEGFA*, *ADM*, *PAI1* and *LOX*, their induction in HIF2α-KO cells was significantly inhibited (Fig. 1a). Next, we studied the effect of HIF2α-KO on epithelial morphogenesis. Control and HIF2α-KO cells were seeded into 3D BME gels and initially grown in normoxic conditions for 1 h, after which half of the cultures were transferred to hypoxia (1% O_2_) and cultured for 6 days. Under normoxic conditions, control cells efficiently formed cysts with a polarized lumen (69.5%), while the majority (57.9%) of HIF2α-KO cells formed multilumen cysts as was also observed for the HIF2α-KD cells (Fig. 1b,c and Fig. S1c,d). A polarized central lumen in HIF2α-KO cells was formed in only 42.1% of the cysts. These data confirmed that loss of HIF2α-expression in normoxia leads to a multilumen phenotype in MDCK cysts. In contrast, loss of HIF2α under hypoxic conditions did not significantly change the morphology of the cysts, most of which lacked a proper organized lumen and displayed an irregular podocalyxin staining pattern as described previously^24^. A central lumen was observed in 23.8% of control cysts and 19.9% of HIF2α-KO MDCK cysts (Fig. 1b,c). Although apical lumen morphogenesis was affected, apical brush border and podocalyxin staining appeared polarized (Fig. 1b and Fig. S2a). However, E-cadherin-stained AJs appeared wider in HIF2αKO cysts (Fig. S2b). Tight junction (TJ) marker ZO-1 localized mostly at the subapical domains of HIF2αKO cyst lumens but in addition we noticed punctate ZO-1-positive vesicles that were more numerous when compared with control cells (Fig. S2b). This data suggests that, unlike HIF1α, HIF2α only plays a minor role, if any, in the hypoxia-induced loss of MDCK cyst polarity. However, HIF2α is needed for efficient cystogenesis of MDCK cells in normoxia and it seems to regulate formation of cell–cell junctions.

### Depletion of HIF2α in MDCK cells leads to changes in the expression levels of adherens junction proteins

**Figure 2:**
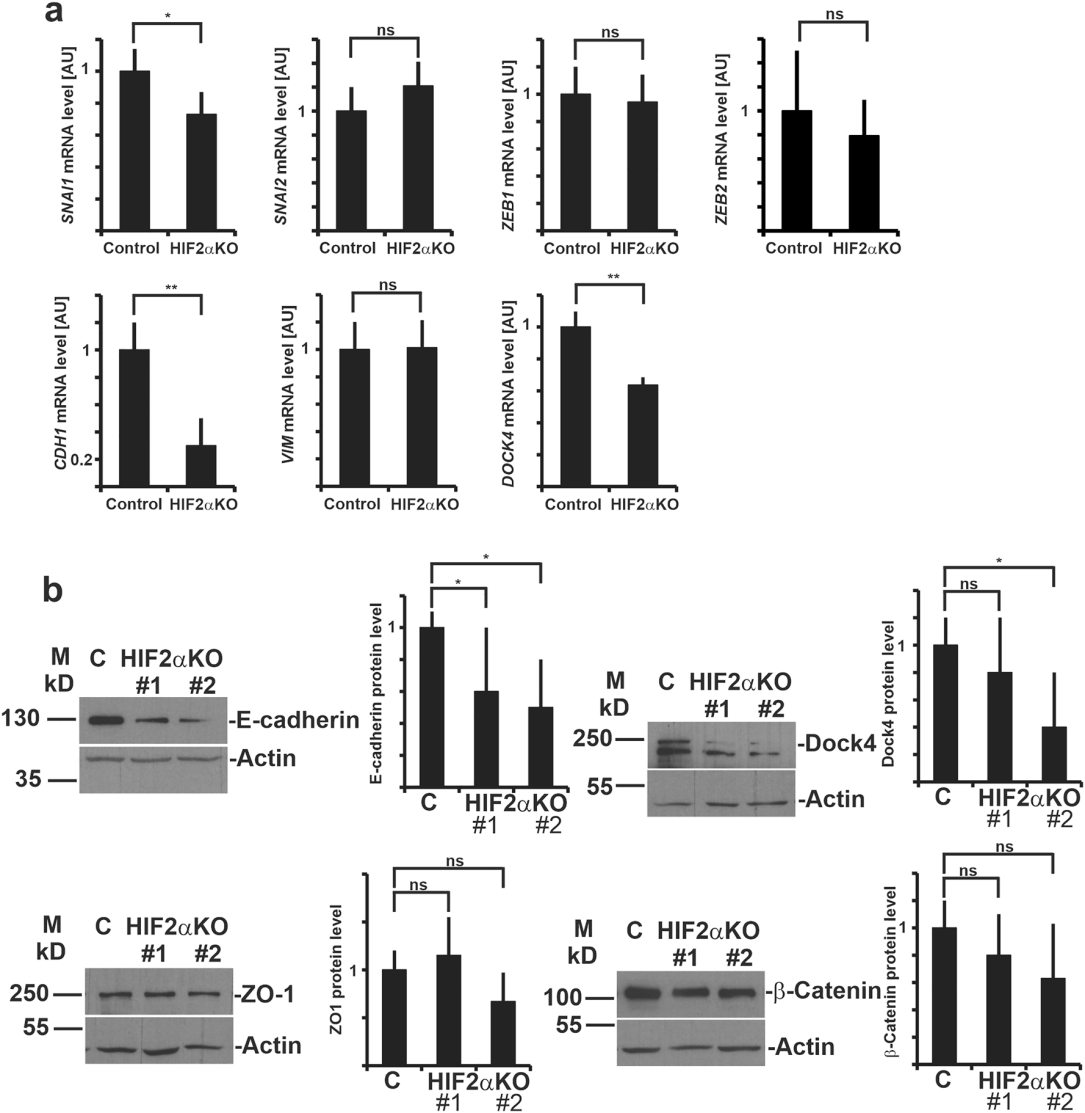
Expression analysis of genes involved in EMT and AJs in HIF2α-KO MDCK cells. (**a**) RT-qPCR analysis of the mRNA expression levels of genes encoding SNAI1, SNAI2, ZEB1, ZEB2, E-cadherin, Vimentin, and Dock4 in control and HIF2α-KO#1 and HIF2α-KO#2 cell lines in normoxic conditions. Relative expression levels are shown mean ± SD, n ≥ 4, expression levels in control cells were normalized to 1, *P < 0.05; **P < 0.001; ns, not significant (unpaired Student’s t-test). (**b**) Western blot analysis of E-cadherin, Dock4, ZO-1 and β-Catenin in control and HIF2α-KO#1 and HIF2α-KO#2 cell lines in normoxic conditions. Actin is shown as a loading control. (**c**) Quantitation of the Western blot data represented in b. Data is shown as mean ± SD (n = 3) of the ratio of protein expression level in HIF2α-KO#1 and HIF2α-KO#2 cell lines relative to control. *P < 0.05; *ns* not significant (unpaired Student’s t-test).

HIF-pathway has been implicated in hypoxia-induced EMT, thus the term “hypoxia-induced EMT” has been proposed^19^. EMT is a major contributor to the development of renal fibrosis and is characterized by the disassembly of cell–cell contacts such as E-cadherin-based AJs^30,31^. The main transcription factors which regulate EMT are the Snail family zinc finger 1 and 2 (*SNAI1* and *SNAI2*) and the zinc finger E-box-binding homeobox 1 and 2 (*ZEB1* and *ZEB2*)^30^. It has been suggested that HIF2α can promote expression of EMT-associated transcription factors in Ras-transformed lung tumors^32^. HIF2α-mediated upregulation of Snail1 was also reported in melanoma cells^33^. EMT is typically determined by two markers, downregulation of E-cadherin and upregulation of vimentin, this regulation may occur at transcriptional and/or post-translational level^30^. To study if EMT-pathway contributes to the cystogenesis failure in HIF2α-KO MDCK cells in normoxia, we examined the expression of the main EMT transcription factors in control and HIF2α-KO cells. A RT-qPCR analysis of the most important EMT transcription factor genes revealed that knockout of HIF2α modestly reduced the mRNA levels of *SNAI1* but did not have significant effect on the levels of *SNAI2*, *ZEB1* or *ZEB2* (Fig. 2a). E-cadherin was significantly downregulated at both mRNA (*CDH1*, Fig. 2a) and protein levels (Fig. 2b), while the other central EMT-marker vimentin showed no change at its mRNA levels (*VIM*) in HIF2α-KO cells (Fig. 2a). The protein levels of β-catenin, a key E-cadherin interaction partner that connects AJs to the actin cytoskeleton but also can act as transcriptional regulator^34^, showed a trend for downregulation but this was not statistically significant (Fig. 2b). ZO-1 proteins organize TJs and are involved in E-cadherin-mediated signaling to regulate actin cytoskeleton^35^. No change was observed for ZO-1 protein levels in HIF2α-KO cells (Fig. 2b).

To screen for potential signaling pathways involved in the HIF2α-dependent regulation of the morphogenetic changes in HIF2α-depleted MDCK cells, a microarray-based gene expression analysis in control and HIF2α-KD#1 MDCK cells grown under normoxic conditions was performed (Gene Expression Omnibus accession number GSE121325). However, the analysis did not reveal clear gene sets significantly associated with known signaling pathways. Since E-cadherin and β-catenin play crucial role in organization of adherent junctions^35^ and E-cadherin expression levels were significantly changed at both transcriptional and post-translational level, we then focused our attention to genes associated with adherent junctions. Joint analysis of microarray data and gene expression profiling interactive analysis (GEPIA) identified Dock4, a GEF for Rac1 and Rap1, as a potential HIF2α-regulated candidate gene as it was found to be co-expressed with the HIF2α gene *EPAS1/HIF2A* (hereafter referred to as *HIF2A*) (http://gepia.cancer-pku.cn/detail.php?gene=EPAS1###). The correlation coefficient between *HIF2A* and *DOCK4* expression in different types of cancers according to GEPIA was up to 0.7 (p = 0; Fig. S3), which demonstrates a significant correlation. Transcriptomic investigations of the clinical database of various human tissues also showed high correlation between *HIF2A* and *DOCK4* expression in many tissues (Fig. S3).

HIF signaling plays a critical role in kidney functions and MDCK is a kidney-derived cell line that represents an epithelial cell lineage with properties resembling mostly the collecting duct epithelium but having also some features of distal tubular cells^36,37^. To investigate *HIF2A* and *DOCK4* co-expression in the kidney in more detail, we analyzed single cell sequencing data from mouse and human kidneys focusing on cell types displaying AJs. We found that *HIF2A* and *DOCK4* are co-expressed in collecting duct and proximal tubular epithelial cells as well as in kidney endothelial cells (Fig. S4). Interestingly, RT-qPCR analysis revealed significant downregulation of *DOCK4* both at mRNA (Fig. 2a) and protein (Fig. 2b) levels in HIF2α-KO cells suggesting that these two genes are not only co-expressed but *DOCK4* expression levels are also regulated by HIF2α. Thus, our analysis revealed robust downregulation of both E-cadherin (*CDH1*) and Dock4 (*DOCK4*) in normoxic HIF2α-KO cells.

### The depletion of Dock4 expression leads to aberrant cyst morphogenesis and decreased levels of adherens junction proteins in normoxia

**Figure 3:**
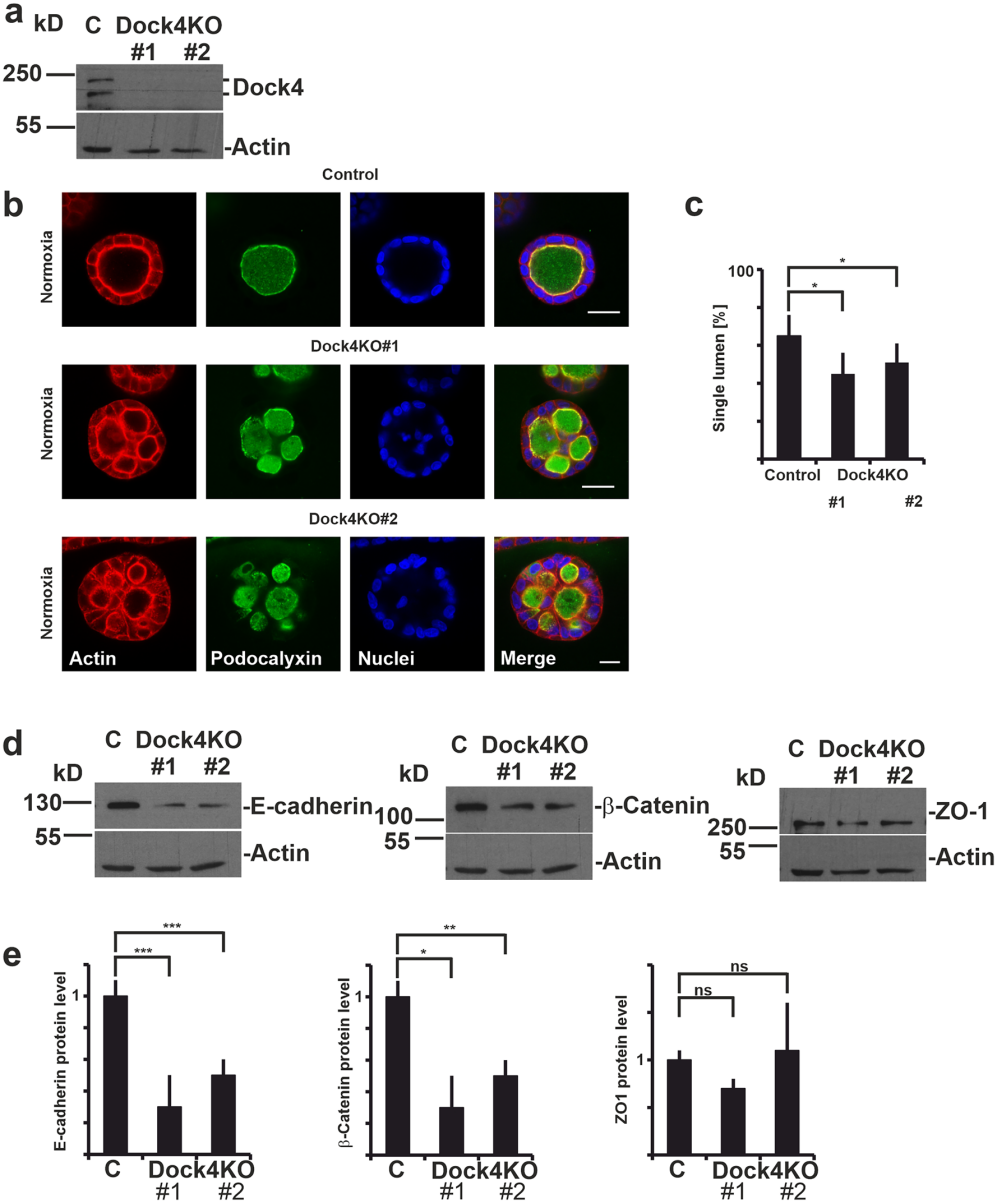
Knockout of Dock4 disrupts morphogenesis of MDCK cysts in normoxic conditions. (**a**) Western blot analysis of Dock4 in control and the Dock4-KO#1 and Dock4-KO#2 MDCK cell lines in normoxic conditions. Actin is shown as a loading control. (**b**) Control and Dock4-KO MDCK cysts were grown in 3D under normoxic conditions. At day 6 the cysts were fixed and stained for an apical membrane protein podocalyxin (green), actin cytoskeleton (red) and nuclei (DAPI, blue). A single confocal slice from the middle of the cysts is shown. Scale bars: 20 µm. (**c**) Quantitation of the cyst phenotypes in control and Dock4-KO #1 and #2 MDCK cell lines grown as in (**b**). The percentage of cysts with single lumen were calculated and averaged from 4 to 8 independent experiments. A minimum of 160 cysts per sample was scored in each experiment. Data are presented as mean ± SD, n ≥ 4. *P < 0.05 (Fischer’s exact test). (**d**) Western blot analysis of E-cadherin, β-Catenin and ZO-1 in control, Dock4-KO#1 and Dock4-KO#2 MDCK cell lines in normoxic conditions. Actin is shown as a loading control. (**e**) Quantitation of the Western blot data in d for E-cadherin, β-Catenin and ZO-1 protein levels. Data are presented as mean ± SD (n = 3) of the ratio of protein expression level in the Dock4-KO#1 and Dock4-KO#2 MDCK cell lines relative to control. *P < 0.05; **P < 0.001; ***P < 0.0001; *ns* not significant (unpaired Student’s t-test).

To investigate the functional role of Dock4 GTPase in MDCK cyst morphogenesis, we designed two guide RNA (gRNA) constructs targeting the sixth exon of canine Dock4 (Table S2) and generated two independent Dock4-KO clones (Table S3) as described previously^29^. Successful editing of both alleles leading to premature stop codon was confirmed by sequencing in each of the clones (Table S3). Depletion of Dock4 expression levels was confirmed by Western blotting (Fig. 3a). MDCK cells transfected with vector containing an empty gRNA-cassette were used as a control in all subsequent experiments.

Control and Dock4-KO cells were seeded into 3D BME gels and grown in normoxic conditions as described above for HIF2α-KO cells. The majority of control cells (64.5%) formed hollow cysts with a single central lumen, while significant reduction of single lumen cysts was observed for Dock4-KO cells (47.7%) (Fig. 3b,c). The major phenotype of the Dock4-KO cysts was formation of multiple lumens (Fig. 3b,c). Thus, depletion of Dock4 led to similar morphological defects in MDCK cyst formation as was observed for HIF2α-KO cells. Moreover, similar to HIF2α-KO cells, examination of junctional proteins in Dock4-KO cells showed downregulation of E-cadherin and β-catenin but no effects on ZO-1-protein levels (Fig. 3d,e). Interestingly, Dock4 mutations have been suggested to cause disruption of AJs to promote tumorigenesis of epithelial cancers^38^. It is thus possible that depletion of Dock4 perturbs cystogenesis by interfering with AJ maturation.

### Dock4 and HIF2A knockout in MDCK cells reduce EGF-induced activation of Rac1/Pak1-signaling

**Figure 4:**
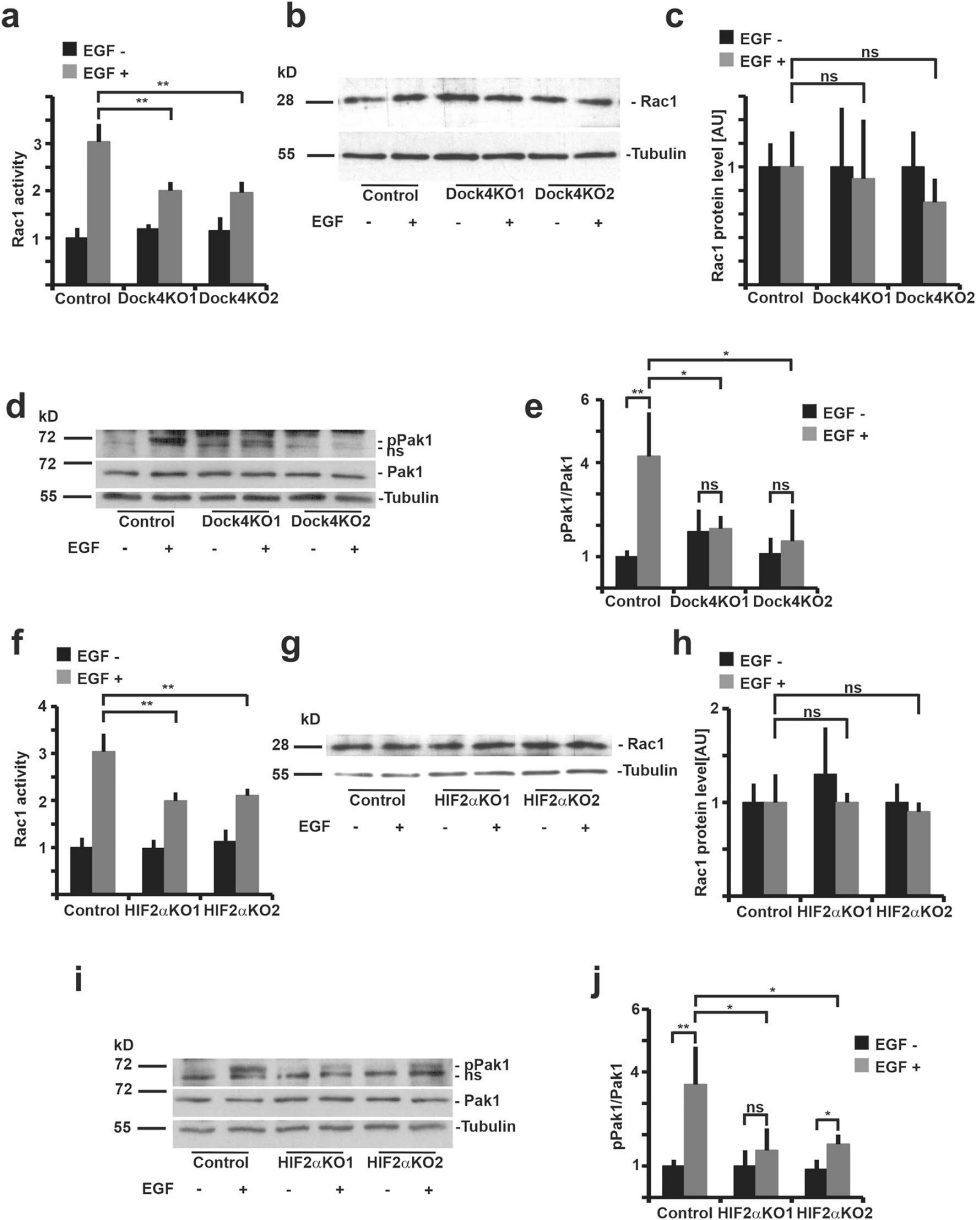
Knockout of Dock4 decreases EGF-induced Rac1/Pak1 activation. (**a**–**c**) Control, Dock4-KO#1 and Dock4-KO#2 MDCK cells were serum starved for 24 h and stimulated with EGF (100 ng/ml) for 10 min. Cells were lysed, and Rac1 activity was detected by G-LISA Rac1 activation assay. (**b**) Rac1 protein levels were measured by Western blotting. Tubulin is shown as a loading control. (**c**) Quantitation of Western blot data of (B) Rac1 protein level. Data are presented as mean ± SD (n = 3) of the ratio of protein expression level in the Dock4-KO#1 and Dock4-KO#2 MDCK cell lines relative to control. **P < 0.001; *ns* not significant (two-way ANOVA). (**d**) Western blot analysis for p-Pak1 and total-Pak1 following EGF stimulation is shown in control, Dock4-KO#1 and Dock4-KO#2 MDCK cells. A representative blot of three independent experiments is shown. (**e**) Quantitation of the data are shown as the ratio of arbitrary absorption units of p-Pak1 and Pak1 (mean ± SD). *P < 0.05; **P < 0.001; *ns* not significant (two-way ANOVA). (**f**) Control, HIF2α-KO#1 and HIF2α-KO#2 MDCK cells were serum-starved for 24 h and stimulated with EGF (100 ng/ml) for 10 min. Cells were lysed, and Rac1 activity was detected by G-LISA Rac1 activation assay. (**g**) Rac1 protein levels were measured by Western blotting. Tubulin is shown as a loading control. (**h**) Quantitation of Western blot data of Rac1 protein levels as shown in g. Data are presented as mean ± SD (n = 3) of the ratio of protein expression level in the HIF2α-KO#1 and HIF2α-KO#2 MDCK cell lines relative to control. **P < 0.001; *ns* not significant (two-way ANOVA). (**i**) Western blot analysis for p-Pak1 and total-Pak1 following EGF stimulation is shown in HIF2α-KO#1 and HIF2α-KO#2 MDCK cells and control cells. A representative blot of three independent experiments is shown. (**j**) Quantitation of the p-Pak1 and total Pak1 levels are shown as the ratio of arbitrary absorption units of p-Pak1 and Pak1 (mean ± SD). *P < 0.05; **P < 0.001; *ns* not significant (two-way ANOVA).

Previous studies on Dock4 have demonstrated its ability to activate Rac1 to regulate AJ stability^39,40^. Importantly, Dock4-mediated Rac1 activation was found necessary for stabilizing AJs and for proper apical lumen formation in endothelial vessels^41^. Next, we studied whether Dock4 acts as a Rac1 GEF downstream of HIF2α activation. To assess Rac1 activation serum-starved control and Dock4-KO MDCK cells were stimulated with EGF followed by a measurement of Rac1 activity. Dock4-KO cells showed significant inhibition of EGF-induced activation of Rac1 (Fig. 4a), while the expression levels of Rac1 protein were not changed (Fig. 4b,c). Next, we determined the activity of Pak1, a well-known downstream effector of Rac1 signaling pathway^42^. In line with Rac1 activity, Pak1 phosphorylation, but not Pak1 total levels, was strongly reduced in EGF-induced Dock4-KO cells when compared with controls (Fig. 4d,e). To confirm the involvement of HIF2α in this pathway we performed a similar analysis using the HIF2α-KO MDCK cells. As shown in Fig. 4f, HIF2α-KO cells showed significantly inhibited Rac1 activation upon EGF-treatment (Fig. 4f–h) as well as reduction of Pak1 phosphorylation (Fig. 4i,j). ZINC69391 is a pharmacological inhibitor of Rac1 which binds to the Dock GEF binding site of Rac1 and therefore functions as a specific inhibitor of Dock/Rac1 signaling^43^. Treatment of control MDCK cells with ZINC69391 inhibited EGF-induced Rac1 activity (Fig. S5a–c) leading to decreased Pak1 phosphorylation (Fig. S5d,e). Taken together, these data suggest that HIF2α-mediated upregulation of Dock4 leads to activation of the Rac1/Pak1 signaling cascade.

### Loss of HIF2a or Dock4 expression affects maturation of adherens junctions in nascent cell–cell contacts

**Figure 5:**
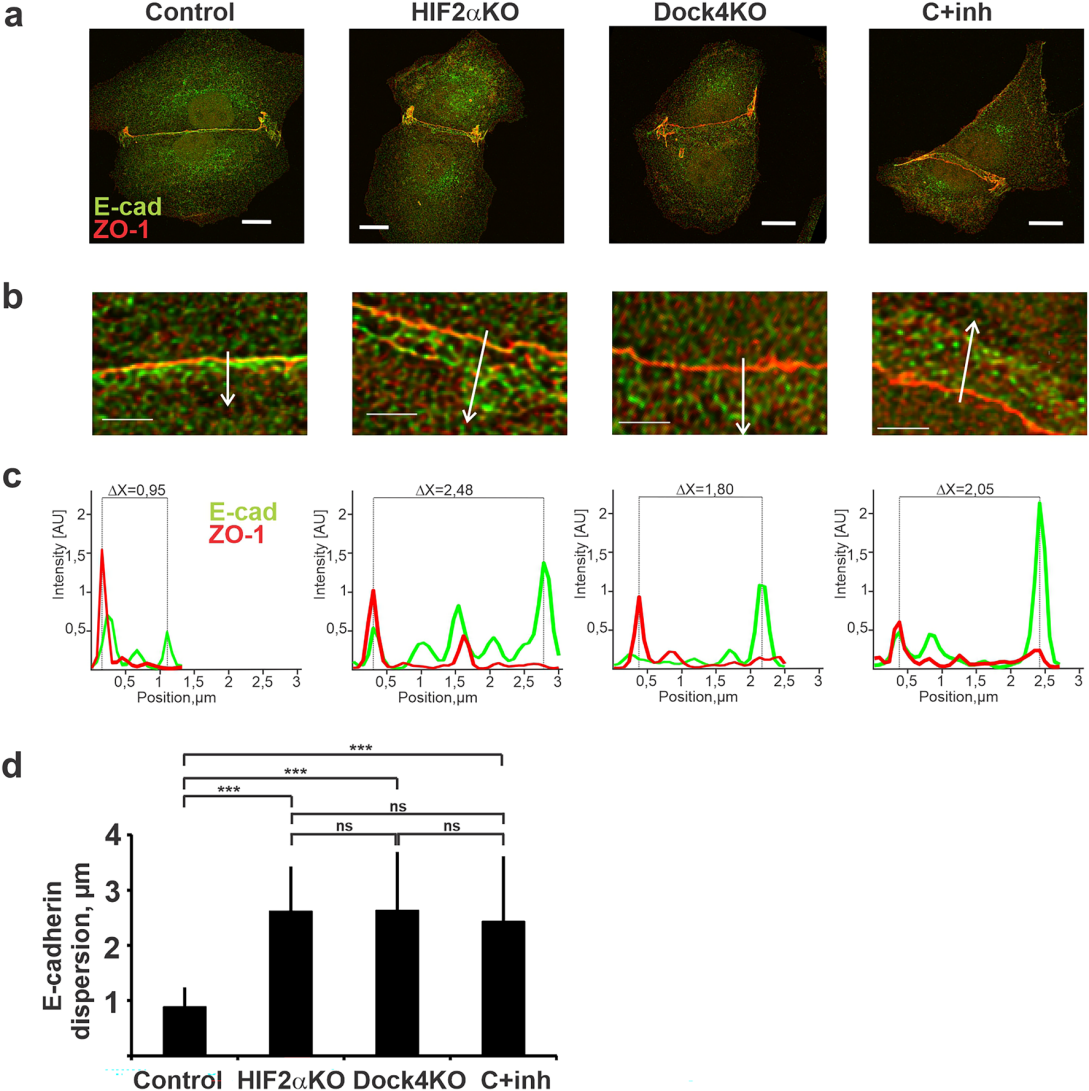
Depletion of HIF2α and Dock4 expression affects formation of nascent cell–cell adhesion contacts. Control, HIF2αKO and DOCK4KO cells were seeded sparsely onto coverslips and grown for 48 h. Rac1/Dock-inhibitor (ZINC69391) was added after the first 24 h when indicated. After a total of 48 h cells were fixed and stained for E-cadherin (green) and ZO-1 (red). Maximum intensity projections of confocal z-stack images are shown (**a**,**b**). Scale bars: 10 µm in a and 2 µm in (**b**). The arrows in b indicate the locations of for intensity histograms shown in (**c**). (**c**) The representative intensity histogram analysis of E-cadherin (green) with ZO-1 (red) from (**b**). (**d**) Quantification of the distribution of intensity peaks of E-cadherin in control, HIF2αKO, DOCK4KO and ZINC69391-treated control cells. Combined data from more than 20 images per sample is shown. Five measurements were made from each maximum intensity profile. Relative width of E-cadherin distribution is shown relative to that of untreated control (normalized to 1). Data is presented as mean ± SD, *, P < 0.05, **, P < 0.001, ***, P < 0.0001 (two-way ANOVA).

Homophilic cadherin-cadherin interactions at AJs connect the plasma membranes of neighboring cells^44^. The cytoplasmic tails of cadherins interact with catenins, which link to the actin cytoskeleton. AJs are highly dynamic structures which, despite mediating relatively stable cell–cell interactions, are under constant remodeling. Loosening or loss of cell–cell contacts leads to disorganization of epithelial tissue architecture^45^. Junctional maturation is a stepwise process. Upon cell–cell contact primordial AJ complexes are formed before the appearance of the TJs and maturation of adjacent subapical AJs below^46–48^. To study the formation and morphology of AJs in more detail we set up an assay to examine the localization pattern of E-cadherin and ZO-1 in newly established cell–cell contacts (Fig. 5). We hypothesized that such early adhesions will readily reveal subtle defects that might not be obvious in more matured AJs^49^. Upon contact, ZO-1 and E-cadherin accumulated at the lateral contact site and compacted into relatively thin stripe (Fig. 5a), presumably driven by lateral actomyosin contractions that are required for TJ and AJ maturation^50,51^. In HIF2α-KO cells the lateral contact membrane appeared tilted and more loosely organized. E-cadherin failed to accumulate into compacted stripe adjacent to ZO-1 staining. To quantify the morphological changes, we performed a distribution analysis of E-cadherin relative to ZO-1 localization (Fig. 5c,d). To address the hypothesis that Dock4 is a critical downstream effector of HIF2α in normoxic MDCK cells, we performed the same analysis of nascent cell–cell junction analysis in Dock4-KO cells and in control cells treated with Dock/Rac1-specific inhibitor (ZINC69391). Similar to HIF2α, both Dock4-KO and ZINC69391-treated cells failed to efficiently compact E-cadherin with ZO-1 (Fig. 5c,d). Our data suggests that HIF2α regulates AJ formation by inducing expression of Dock4 that activates Rac1/Pak1 signaling necessary for efficient AJ maturation and subsequently coordinated formation of apical lumen during MDCK cystogenesis.

## Discussion

In the present work, we identify a novel pathway in kidney epithelial cells where HIF2α functions in normoxic conditions to induce expression of Dock4 that in turn activates Rac1/Pak1 signaling necessary for robust assembly of AJs. Perturbation of this pathway causes formation of multiple lumens in the MDCK cystogenesis assay. Interestingly, HIF2α has been previously reported to regulate AJ formation in endothelial cells in normoxia, although the proposed mechanism was different and involved HIF2α-mediated transcription of vascular endothelial cadherin^52^.

Our data extends the accumulating evidence that HIF2α signaling is significantly different from HIF1α signaling and it operates also in normoxic conditions. While both HIF1α and HIF2α are stabilized in hypoxic conditions and form a dimer with HIFβ to bind to HRE-sites within selected promoters to induce transcription of hypoxia and inflammatory stress response genes they have also non-overlapping target genes^6–9,53^. A partial explanation might be their different stability dynamics. HIF1α is undetectable in normoxia but it rapidly accumulates in hypoxia and its levels swiftly fall back following reoxygenation. HIF2α levels are induced more slowly in hypoxia, and it also takes longer for HIF2α levels to be downregulated. In light of the present work, and unlike HIF1α, HIF2α appears to be expressed under modest hypoxia or even normal physiological oxygen levels^6,16,18^.

HIFs have also been reported to function via HRE-independent mechanisms. HIF2α was shown to activate IL-31 via a HIF1β-independent mechanism^54^. Recently, HIF1β-independent HIF2α target genes were also identified in neuroblastoma cells^18^. Moreover, HIF1α was stabilized under normoxic conditions by T-cell receptor stimulation or IL‐6-mediated STAT signaling^55,56^. Curiously, one of the DOCK family members, DOCK8 was reported to function as a negative regulator of HIF2α nuclear translocation in CD4 + T cells^54^. Whether Dock4 activation may similarly function to provide negative feedback signaling for HIF2α remains to be studied. HIF2α itself is a direct target of GATA-3 and STAT6 transcription factors^57,58^.

The HIF2α/Dock4/Rac1/Pak1 signaling cascade identified in this study provides potential links between HIF2α-signaling and tumorigenesis. The main function of Dock proteins is to regulate cell motility, polarity, adhesion and cytoskeletal organization via activation of Rac1 and Cdc42. Dock4 is highly expressed in the brain and it regulates spine formation through Rac activation^59^. Dock4/Rac1-signaling has been implicated in the regulation of β-catenin stability and activation of the Wnt-pathway^60^. Importantly, β-catenin is also a critical component of AJs, that together with α-catenin regulates AJ interactions with the actin cytoskeleton. By using superresolution microscopy, Zaidel-Bar and coworkers reported that E-cadherin forms nanometer scale domains that upon cell–cell contact, accumulate subapically at the lateral membranes in actin-dependent manner to form mature AJs^61^. It is tempting to speculate that HIF2α-Dock4-Rac1-signaling axis stabilizes β-catenin and regulates actin dynamics thereby driving subapical clustering of E-cadherin domains. Dock4 mutations or depletion have been observed in ovarian and prostate cancers and were shown to contribute to tumorigenesis^38^. Dock4-mediated activation of Rap1 GTPase was shown to promote stability of N-cadherin mediated cell–cell junctions in osteosarcoma cells^38^. We report here a similar function for Dock4-mediated activation of Rac1 in kidney epithelial cells. In breast cancer cells Dock4 localizes at the tips of membrane protrusions where it promotes cell migration via activation Rac1^39,40,62^.

HIF2α is commonly thought to be upregulated in tumor cells^15^. However, our analysis of the *HIF2A* expression profile across all tumor samples and paired normal tissues show that *HIF2A* expression levels vary depending on the tumor type (http://gepia.cancer-pku.cn/detail.php?gene=EPAS1###). In lung adenocarcinoma (LUAD) and lung squamous cell carcinoma (LUSC) *HIF2A* expression is 8- to 15-fold higher in normal tissue than in cancer samples. In invasive carcinomas of the breast (BRCA), thyroid carcinoma (THCA), uterine corpus endometrial carcinoma (UCEC) and uterine carcinosarcoma (UCS) *HIF2A* expression is 3- to ninefold more expressed in normal than in cancer samples. These findings are in line with our data where loss of HIF2α in MDCK cells led to disrupted epithelial morphogenesis. However, analysis of kidney renal clear cell carcinoma (KIRC) database samples showed twofold higher *HIF2A* expression in cancer than in normal samples (http://gepia.cancer-pku.cn/detail.php?gene=EPAS1###). Canonical HIF signaling is an important driver of renal cancers and it is thus possible that HIF2α activation is crucial for maintaining the hypoxia response signaling promoting cancer progression in the kidney. In most other tumor types HIF2α might not be essential for growth and loss of HIF2α could directly promote tumorigenesis. Further studies are needed to characterize the context-specific factors that determine the role of HIF2α in tumorigenesis.

We used MDCK cyst formation, a classical model to study cell polarization to address the role of HIF2α in kidney epithelia. Using canine cell model is not without challenges, we could not use established antibodies to detect human HIF2α since these did not cross-react with canine HIF2α. Recently, human kidney organoid cultures have become a feasible alternative model. It will be important to validate our results in such human organoid models in the future.

## Methods

### Cell culture and reagents

MDCK strain II cells (ATCC: CCL-34) were cultured for 2D and 3D culture as described previously^27^. MDCK cells were cultured either in normoxic (16% O_2_, 5% CO_2_, and 79% N_2_) or hypoxic (1% O_2_, 5% CO_2_, and 94% N_2_) conditions in an Invivo2 400 hypoxic workstation (Baker Ruskinn, Sanford, Maine, USA). The cells were regularly tested, every 6 months, to be mycoplasma-free. ZINC69391 (AOB6643, AOBIOUS), a selective inhibitor of Rac1 was reconstituted in dimethyl sulfoxide (DMSO) and used at a concentration of 100 µM.

### Generation of HIF2α-KD cells by retrovirus-mediated RNA interference

HIF2α-KD cell lines were generated by infection of MDCK cells with retroviruses coding for shRNA constructs, and the infected cells were selected with puromycin as previously described^25^. Three targeting sequences were cloned into RVH1-puro vector and their efficiencies to silence HIF2α expression in MDCK cells were determined by RT-qPCR (Table S1).

### Generation of HIF2α-KO and Dock4-KO cells by CRISPR-Cas9-mediated genome editing

A 20-bp guide sequences targeting the fourth exon of canine *HIF2α* (Epas1)) and the sixth exon of canine *Dock4* were designed online using Zhang’s laboratory web resource (www.genome-engineering.org). gRNA-encoding oligonucleotides (Sigma-Aldrich) were cloned into the vector SpCas9(BB)-2A-GFP (PX458, Addgene plasmid ID 48138) using standard procedures as described^29^. The generation of the HIF2α-KO and Dock4-KO cells via CRISPR-Cas9- mediated non-homologous end-joining (NHEJ) DNA repair and the screening were performed according to described guidelines^29^.

In brief, the MDCK cells were transiently transfected with the genome editing CRISPR-Cas9 construct and 48 h post-transfection cells were subjected to single-cell-sorting. The single-cell clones were expanded and screened for frame-shift mutations; shortly, a region spanning the target site was amplified by PCR from genomic DNA isolated from clonal cell lines. PCR products were subsequently cloned into pUC19 (Invitrogen). 15–20 sequences were selected based on FASTA similarity search-tool (EMBL-EBI) (Table S2).

### Immunostaining

For immunostaining 3D MDCK cells in 30 µl blobs of Cultrex® 3D Culture Matrix™ BME (3445-005-01, Trevigen, Gaithesburg, Maryland, USA) were cultured in normoxic or hypoxic conditions for 6 days, 2D MDCK cells were cultured in normoxic conditions for 24 h. The immunostaining procedure was performed as described previously^24^. The following antibodies were used: podocalyxin antibody (3F2/D8 cell line, Developmental Studies Hybridoma Bank, Iowa City, Iowa, USA), E-cadherin antibody (rr1, 1:100, Developmental Studies Hybridoma Bank, Iowa City, Iowa, USA) and ZO-1 antibody (339100, 1:200, Invitrogen, Carlsbad, California, USA), all corresponding secondary antibodies were purchased from Abcam (Cambridge, UK), TRITC-phalloidin (P1951, 1:500, Sigma Alrich, St. Louis, Missouri, USA) and DAPI (D9542, 1:500, Sigma Alrich, St. Louis, Missouri, USA).

### RNA isolation and quantitative real-time PCR

2D MDCK cells were cultured in normoxic conditions for 24 h, followed by 24 h in hypoxia or normoxia. Total RNA was isolated from the 2D and 3D cells using RNeasy Kit (Qiagen, Hilden, Germany), according to the manufacturer’s protocol. The total RNA was reverse transcribed into cDNA using an iScript cDNA synthesis kit (Bio-Rad Laboratories, Hercules, California, USA). Quantitative real-time PCR (RT-qPCR) was performed using iTaq Universal SYBR Green Supermix (Bio-Rad Laboratories, Hercules, California, USA) and a CFX96 Touch real-time PCR detection system. Primer sequences are listed in Table S4. Expression levels were normalized to TATA box binding protein.

### Western Blotting

2D MDCK cells were seeded at a density of 7.5 × 10^5^ cells in 10-cm diameter dishes and cultured in normoxic conditions for 24 h and then transferred to hypoxic conditions for 24 h. Proteins were extracted using RIPA lysis buffer (25 mM Tris pH 7.8, 150 mM NaCl, 0.1% SDS, 0.5% sodium deoxycholate, 1% Triton X-100) and SDS-PAGE and western blotting were performed as described previously^24^. Full scans of the blots are shown in Supplementary Fig. S6. The primary antibodies used were as follows Dock4 (ab85723, Abcam), ZO-1 (#339100, Thermo Fisher Scientific), E-cadherin (rr1, Developmental Studies Hybridoma Bank), β-Catenin (#610153, BD Biosciences), Rac1 (ABIN2787398, antibodies-online), Pak1 (sc-166174, Santa-Cruz), P-Pak1 (sc-135755, Santa-Cruz), tubulin (T7941, Sigma Aldrich), β-Actin (NB 600-501, Novus biological).

### Microscopy and image acquisition

Confocal images were acquired at RT using an Olympus FluoView-1000 laser-scanning confocal microscope with 100 × UPlanSApo (NA:1.40) oil immersion objective (Olympus, Tokyo, Japan). Sequential z-stack scans were performed using 405 nm, 488 nm and 543 nm laser lines for fluorophore excitation coupled with DAPI (430–470 nm), GFP (505–525 nm) and Cy3 (560LP) emission filters, respectively. For 3D morphology analysis at least 160 cysts per sample were analyzed and scored for presence of single central hollow lumen delineated in most sample sets by strong podocalyxin staining. For some samples lumens were assessed using actin- (TRITC/Alexa488-Phalloidin) or ZO-1-staining. Cysts with no or multiple small lumens were scored as abnormal. For localization pattern analysis image stacks were deconvolved and analyzed using Huygens Professional software (Scientific Volume Imaging, Hilversum, Netherlands). Five measurements of E-cadherin dispersion per each image maximum intensity projection were performed and their average from 20 or more cells was analyzed. Representative images were collected with the FV10-ASW software (Olympus, Tokyo, Japan) and imported into Adobe Photoshop CS (Adobe Systems, San José, California, USA). Western blot bands were quantified using Image J. Images were imported into Photoshop and CorelDraw for creation of figures.

### RAC1 activation assay

To assess Rac1 activation Rac1 G-LISA kit (Cytoskeleton) was used according to the manufacturer’s protocol. Cells were cultured first for 12 h in minimal essential medium (MEM) (Gibco) containing 5% fetal bovine serum (FBS, Thermo Fisher Scientific) after which they were washed with PBS and cultured for an additional 12 h in MEM without FBS. Then cells were washed with PBS and cultured for an additional 4 h in MEM without FBS with or without adding Rac1 inhibitor ZINC69391 to final concentration of 100 µM. Then cells were washed with PBS and incubated for 5 min with EGF (Peprotech) at concentration 100 ng/ml. Protein was isolated using standard G-LISA buffer GL36 (Tris pH 7.5, MgCl_2_, NaCl, IGEPAL and SDS). Obtained lysate was aliquoted and frozen in liquid nitrogen. The Rac1 G-LISA kit (Cytoskeleton) contains a Rac-GTP-binding protein linked to the wells of a 96 well plate. Active, GTP-bound Rac1 in cell/tissue lysates will bind to the wells while inactive GDP-bound Rac1 is removed during washing steps. The bound active Rac1 is detected with a Rac1 specific antibody. The degree of Rac1 activation is determined by comparing readings from activated lysates versus non-activated lysates. Plates were read in a Multilabel Counter 1420 VICTOR3V (Perkin Elmer, Waltham, Massachusetts, USA) at 450 nm.

### Statistical analysis

Data are expressed as means ± SD of at least three independent experiments. Comparative data were analyzed using the unpaired or paired Student’s *t*-test (two groups), two-way ANOVA (three or more groups) or Fischer’s exact test (cyst phenotype quantitation). Statistical significance is indicated with asterisks; * P < 0.05, ** P < 0.01, *** P < 0.001.

### Supplementary Information

Supplementary Information.

## Data Availability

The microarray data generated during this study has been deposited in the Gene Expression Omnibus database under accession number GSE121325.
