# Supplementary material for: HIF2α-dependent Dock4/Rac1-signaling regulates formation of adherens junctions and cell polarity in normoxia
Source: Sci Rep. 2024 May 27;14:12153. doi: 10.1038/s41598-024-62955-7 (PMC11130225; doi:10.1038/s41598-024-62955-7)
Supplement: Supplementary file 1 — Supplementary Information. [file 41598_2024_62955_MOESM1_ESM.pdf]

Supplementary data

Raykhel *et al*. "*HIF2α-dependent Dock4/Rac1-signaling regulates formation of adherens junctions and cell polarity in normoxia*"

Supplementary Figures S1-S6

Supplementary Tables S1-S4

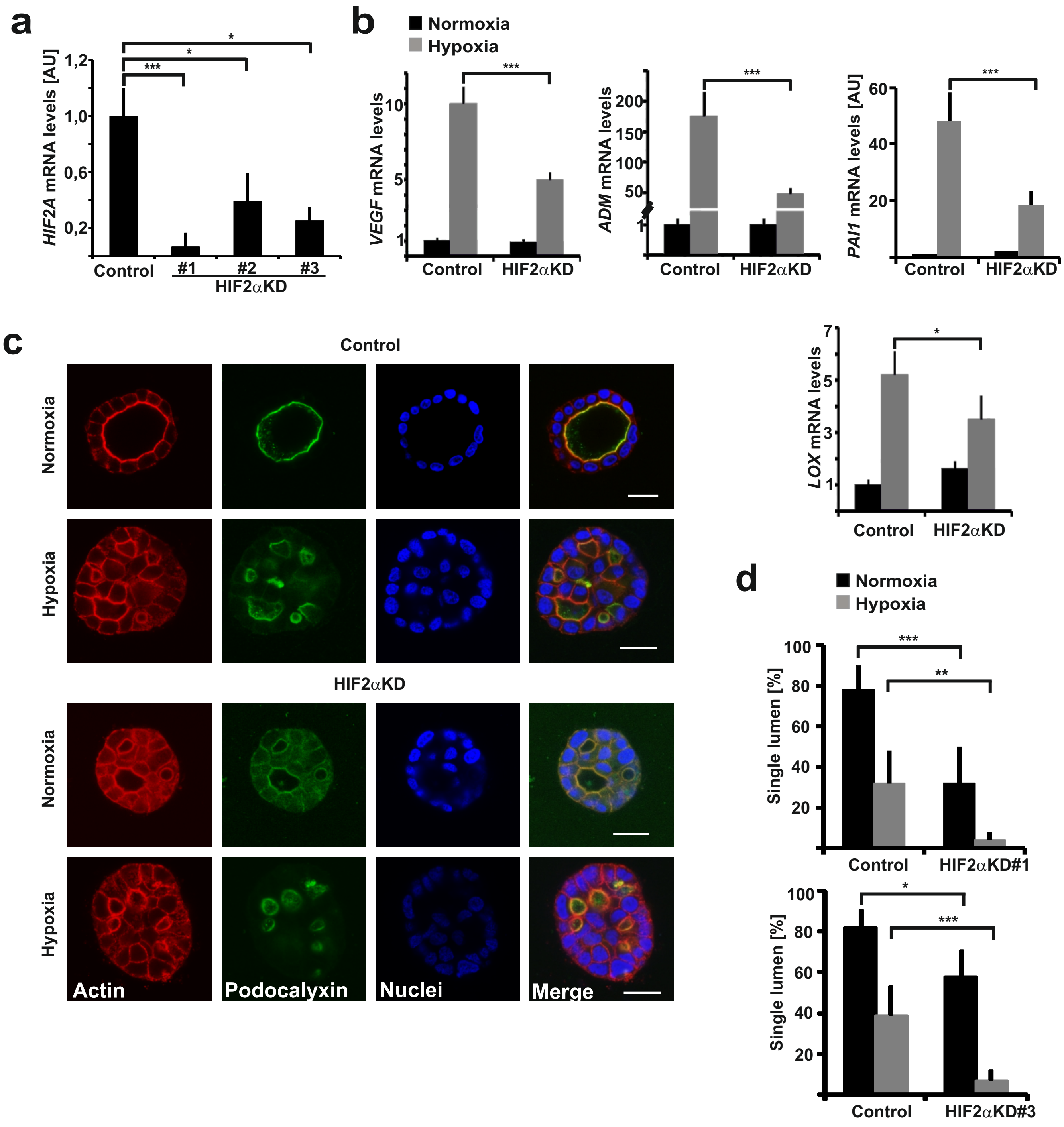

Figure S1. HIF2α-knockdown disrupts morphogenesis of MDCK cysts in normoxic conditions. (a) RT-qPCR analysis of HIF2α mRNA expression levels in control MDCK cells and in three independent HIF2α knockdown (HIF2αKD#1-3) MDCK cell lines. Data are presented as mean ± SD (n ≥ 3). **(b)** RT-qPCR analysis of the mRNA expression level of selected HIF target genes in 2D cultures of control and the HIF2α-KD#1 cell lines under normoxia and hypoxia (48h 1% O2) conditions. Data are presented as mean ± SD (n ≥ 3). *P < 0.05; ***P < 0.0001 (two-way ANOVA). **(c)** Control and HIF2α-KD MDCK cysts were grown in 3D under normoxic or hypoxic (1% O2) conditions. On day 6 the cysts were fixed and stained for apical membrane marker podocalyxin (green), cytoskeletal marker actin (TRITC-phalloidin; red) and nuclear marker (DAPI; blue). A single confocal slice from the middle of each cyst is shown. Scale bars = 20 μm. **(d)** Quantitation of the cyst phenotypes in control, HIF2αKD#1 and HIF2αKD#3 MDCK cell lines. The percentage of cysts with single lumen were calculated and averaged from 4–8 independent experiments. A minimum of 160 cysts per sample was scored in each experiment. Data are presented as mean ± SD, n ≥ 4. **P < 0.001; ***P < 0.0001 (Fischer’s exact test).

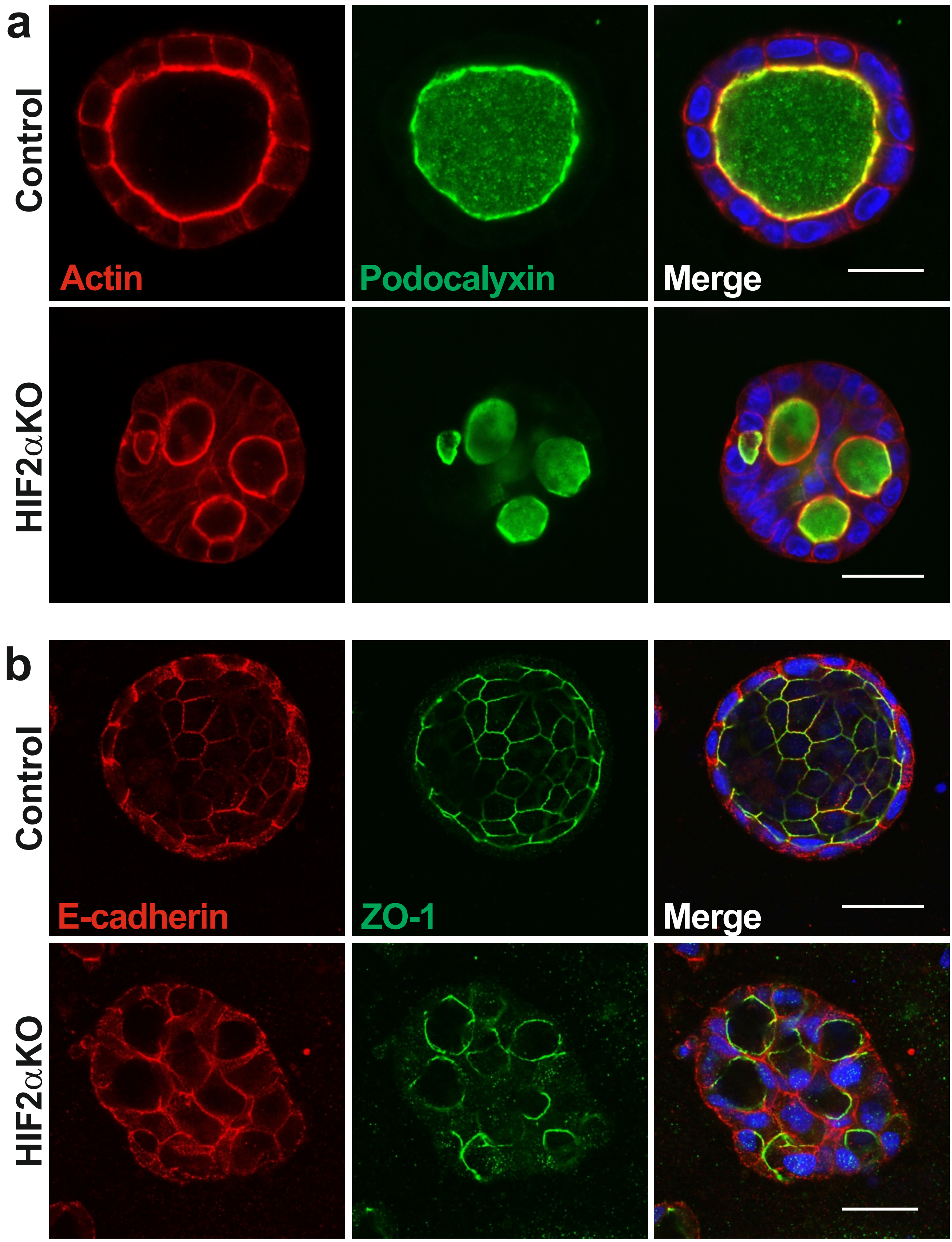

Figure S2. Polarity of apical, adherens junction and tight junction markers in multilumenal *HIF2A*-knockout cysts. Control and HIF2α-KO MDCK cysts were grown in 3D under normoxia. At day 6 the cysts were *fi*xed and stained for **(a)** an apical membrane protein podocalyxin (green) and actin cytoskeleton (TRITC-phalloidin; red) or **(b)** for tight junction marker ZO-1 (green) and a basolateral adherens junction marker E-cadherin (red). In both cases nuclei were stained using DAPI (blue). A single confocal slice from the middle of the cysts is shown. Scale bars: 20 µm.

a

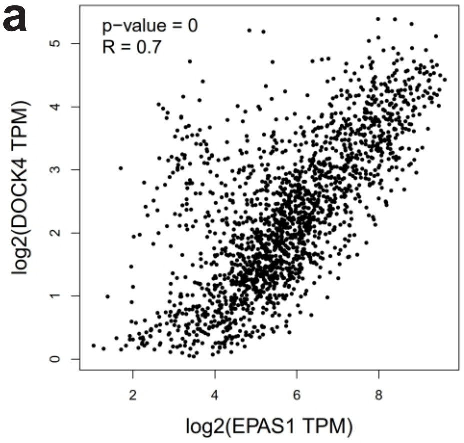

b

	Epas1 - Dock4	
**TCGA(a)Tumor (Cancer name)**	**R**	**P-value**
Uveal Melanoma (UVM)	0,69	1,60E-12
Diffuse large B-cell lymphoma (DLBC)	0,69	1,1E-07
Colon Adenocarcinoma(COAD)	0,68	0
Pancreatic adenocarcinoma (PAAD)	0,59	0
Stomach adenocarcinoma (STAD)	0,53	0
Kidney Renal Clear Cell Carcinoma (KIRC)	0,52	0
Rectum adenocarcinoma (READ)	0,52	8,5E-08
Thymoma (THYM)	0,51	2,9E-09

c

	**Epas1 - Dock4**	
**GTEx(a)**	**R**	**P-value**
Brain - Substantia nigra	0,58	2,20E-06
Colon - Sigmoid	0,6	5,60E-15
Colon - Transverse	0,68	0
Esophagus - Gastroesophageal Junction	0,56	7,40E-13
Esophagus - Mucosa	0,76	0
Heart - Atrial Appendage	0,59	0
Kidney - Cortex	0,71	2,10E-05
Lung	0,56	0
Pituitary	0,77	0
Prostate	0,56	1,20E-09
Minor Salivary Gland	0,77	6,30E-12
Skin - Not Sun Exposed (Suprapubic)	0,61	0
Small Intestine - Terminal Ileum	0,67	4,80E-13
Spleen	0,56	9,30E-10
Stomach	0,77	0
Testis	0,7	0
Vagina	0,56	2,90E-08

Figure S3. Gene Expression Profile Interactive Analysis (GEPIA) transcriptomic investigations of the clinical database showed a high correlation between HIF2α (encoded by the *EPAS1/HIF2A* gene) and *DOCK4* expression in different types of cancers and various human tissues. (a) Correlation between *HIF2A* and *DOCK4* expression in **(b)** 8 different types of cancer patients was analyzed through Gene Expression Profiling Interactive Analysis (GEPIA) (http://gepia.cancer-pku.cn/detail.php?gene=EPAS1###). Result for each type of cancer is presented in B. The Cancer Genome Atlas (TCGA). **(c)** Correlation between *HIF2A* and *DOCK4* expression in different human tissues was analyzed through Gene Expression Profiling Interactive Analysis (GEPIA) (http://gepia.cancer-pku.cn/detail.php?gene=EPAS1###). The Genotype-Tissue Expression (GTEx).

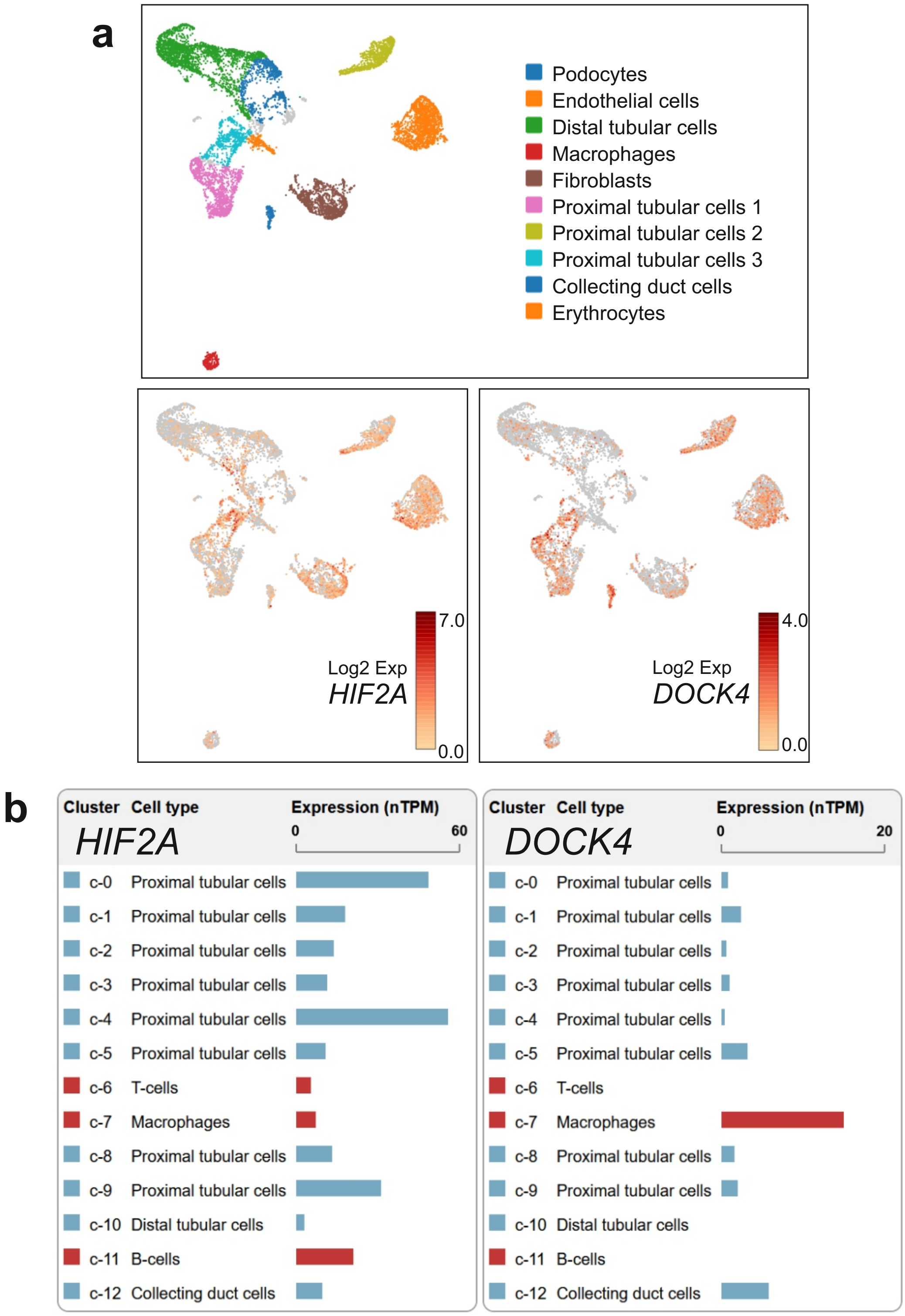

Figure S4. Expression of *HIF2A* (*EPAS1*) and *DOCK4* in kidney cell types. Single-cell transcriptomic datasets from mouse (A) and human (B) kidneys were analyzed for expression of *HIF2A* (*EPAS1*) and *DOCK4*. **(a)** LOUPE raw-file of mouse kidney FFPE tissue singleplex dataset (www.10xgenomics.com) was downloaded and the different cell types were classified using the Loupe Browser (6.2.0). Relative expression levels of *HIF2A* and *DOCK4* in the different cell clusters are shown. In mouse kidney *HIF2A* is abundantly expressed in most cell types with proximal tubular cells and endothelial cells showing high expression levels. The overall levels of *DOCK4* expression are more modest but it is also expressed at significant levels in both proximal tubular cells and endothelial cells. **(b)** *HIF2A* and *DOCK4* expression levels were plotted from human kidney single cell RNA-seq expression dataset (the Human Protein Atlas; www.protein atlas.org). In the human kidney *HIF2A* was highly expressed in multiple cell types. Especially high levels were observed in proximal tubular cells. *DOCK4* levels were more modest with collecting duct epithelial cells expressing the highest levels from E-cadherin-positive cell populations.

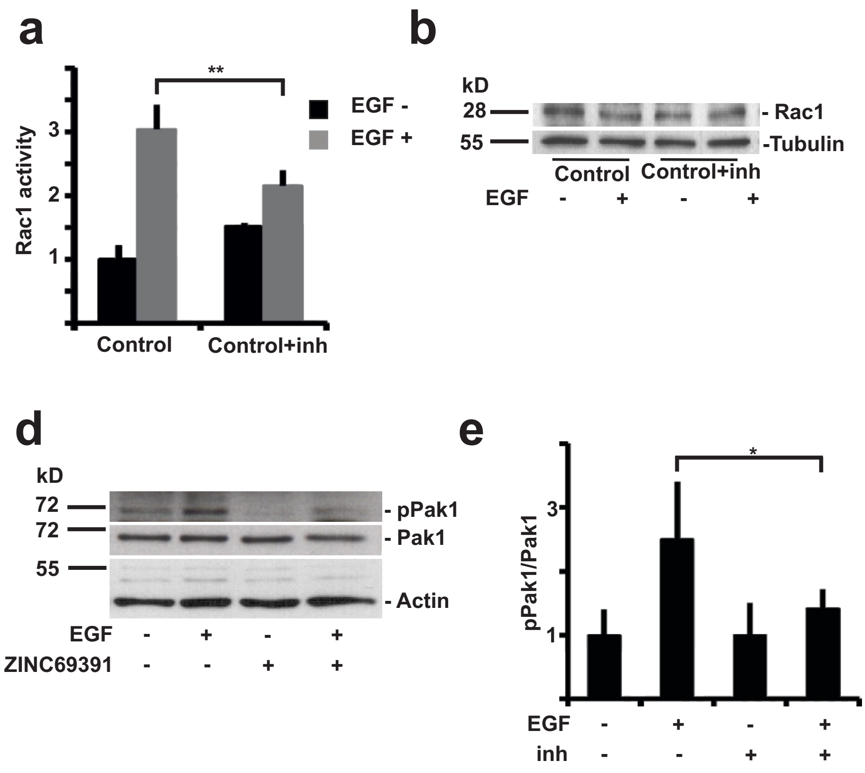

Figure S5. Effect of Rac1 inhibition on Pak1 activation. Control MDCK cells were serum-starved for 24h, pre-treated with Rac1 inhibitor ZINC69391 (100 µM) for 1 h prior to EGF stimulation, stimulated with EGF (100ng/ml) for 10 min. **(a)** Cells were lysed, and Rac1 activity was detected by G-LISA Rac1 activation assay (A). **(b)** Rac1 protein was determined by Western blotting. Tubulin was used as a loading control. A representative blot of three independent experiments is shown. **(c)** Quantitation of the Rac1 protein levels. Data are presented as mean ± SD (n = 3) of the ratio of protein expression levels in Rac1 inhibitor ZINC69391 pre-treated MDCK cell lines relative to control cells. **P < 0.001; ns = not significant (two-way ANOVA). **(d)** Effect of Rac1 inhibition on phosphorylation of Pak1. Western blot analysis for p-Pak1 and total-Pak1 following EGF stimulation is shown in control cells and in cells pre-treated with Rac1 inhibitor ZINC69391. Actin was blotted as a loading control. A representative blot of three independent experiments is shown. **(e)** Quantitation of the combined data from three independent experiments is shown as the ratio of p-Pak1/Pak1 (mean ±SD). *P < 0.05; **P < 0.001; ns = not significant (two-way ANOVA).

Figure 2b

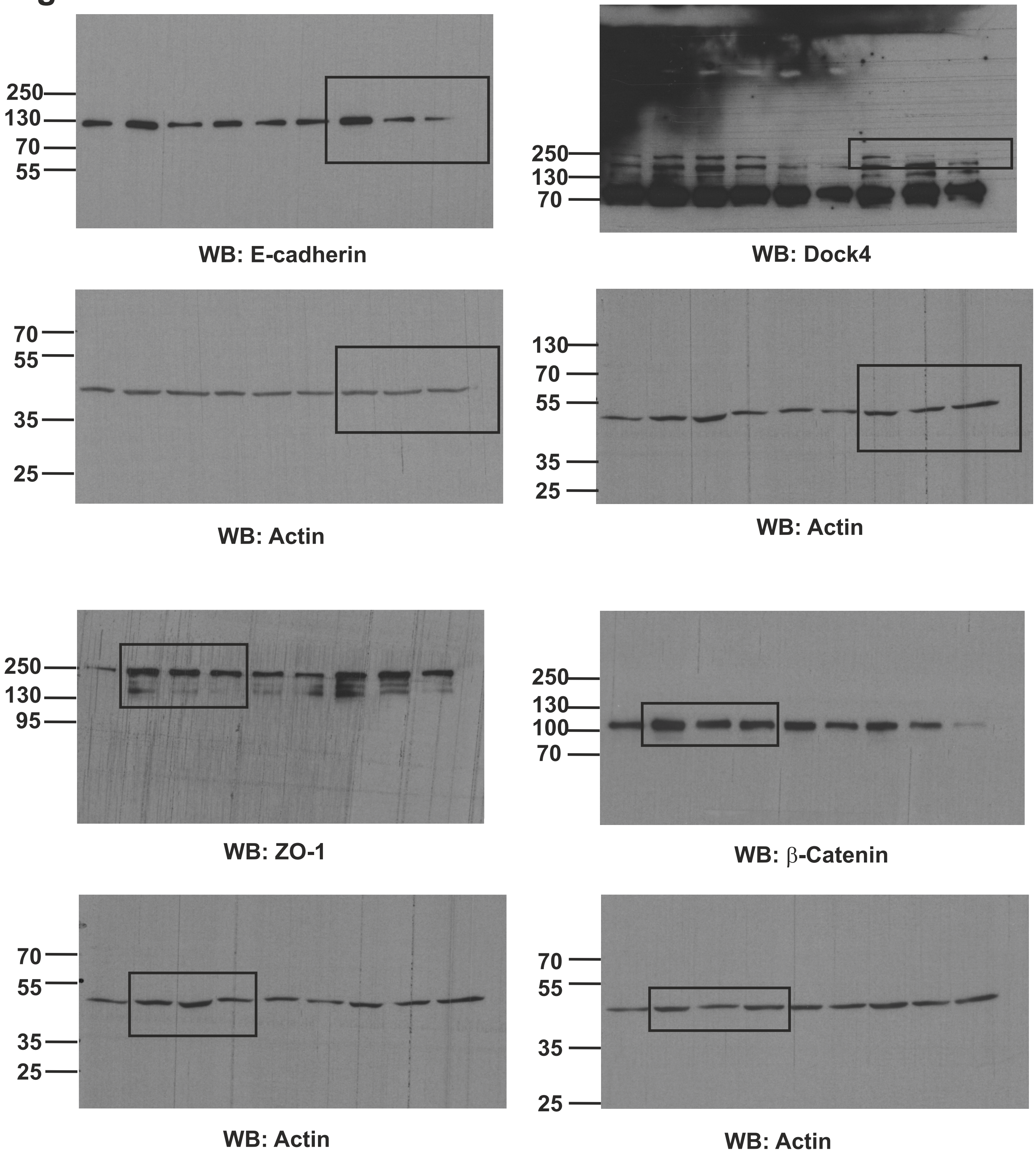

Figure S6. Full scans of the cropped western blots shown in the figures of this study. Page 1/5, continued in the next page...

Figure 3a, d

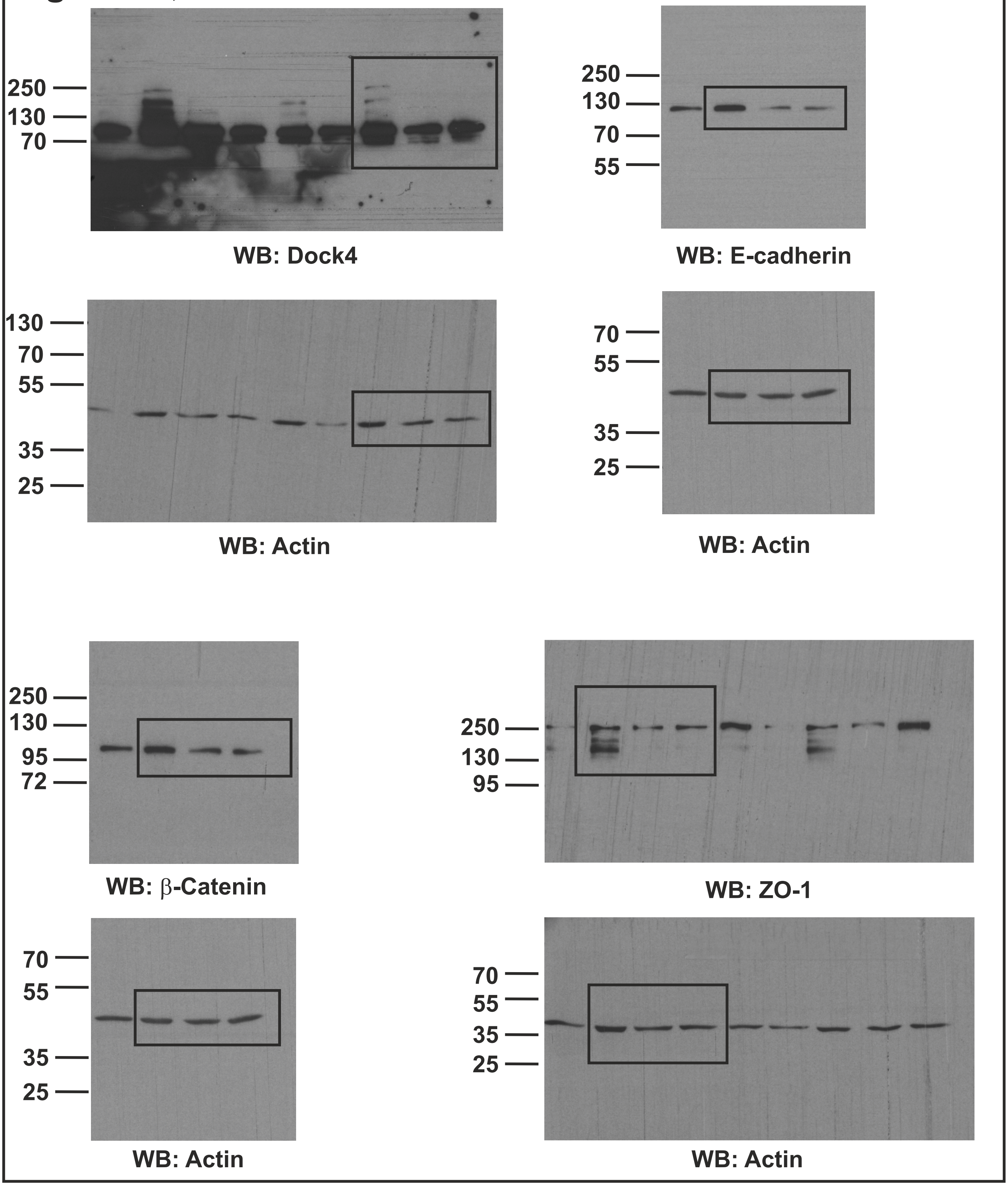

Figure S6. Full scans of the cropped western blots shown in the figures of this study.
Page 2/5, continued in the next page...

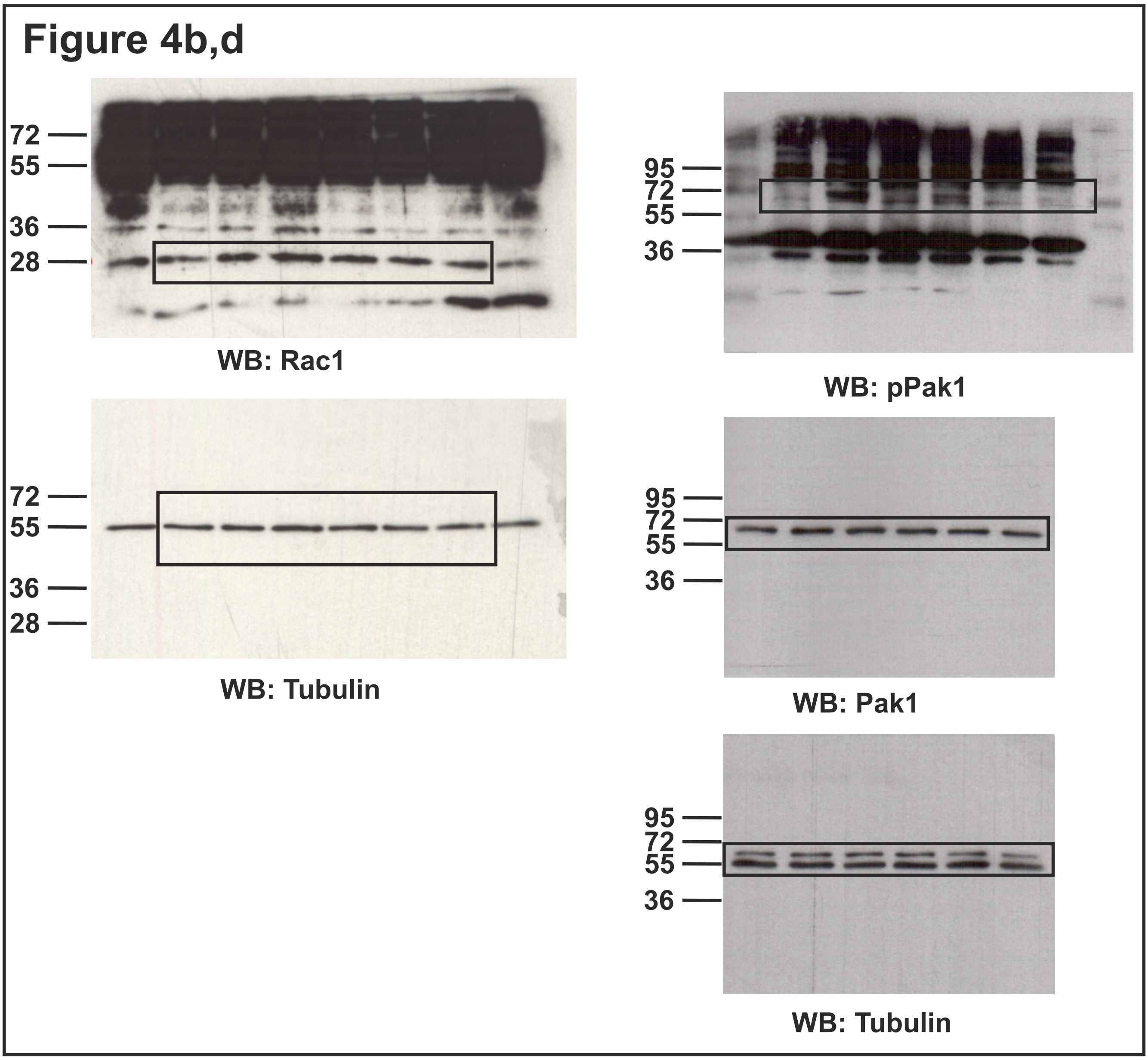

Figure S6. Full scans of the cropped western blots shown in the figures of this study.
Page 3/5, continued in the next page...

Figure 4g,i

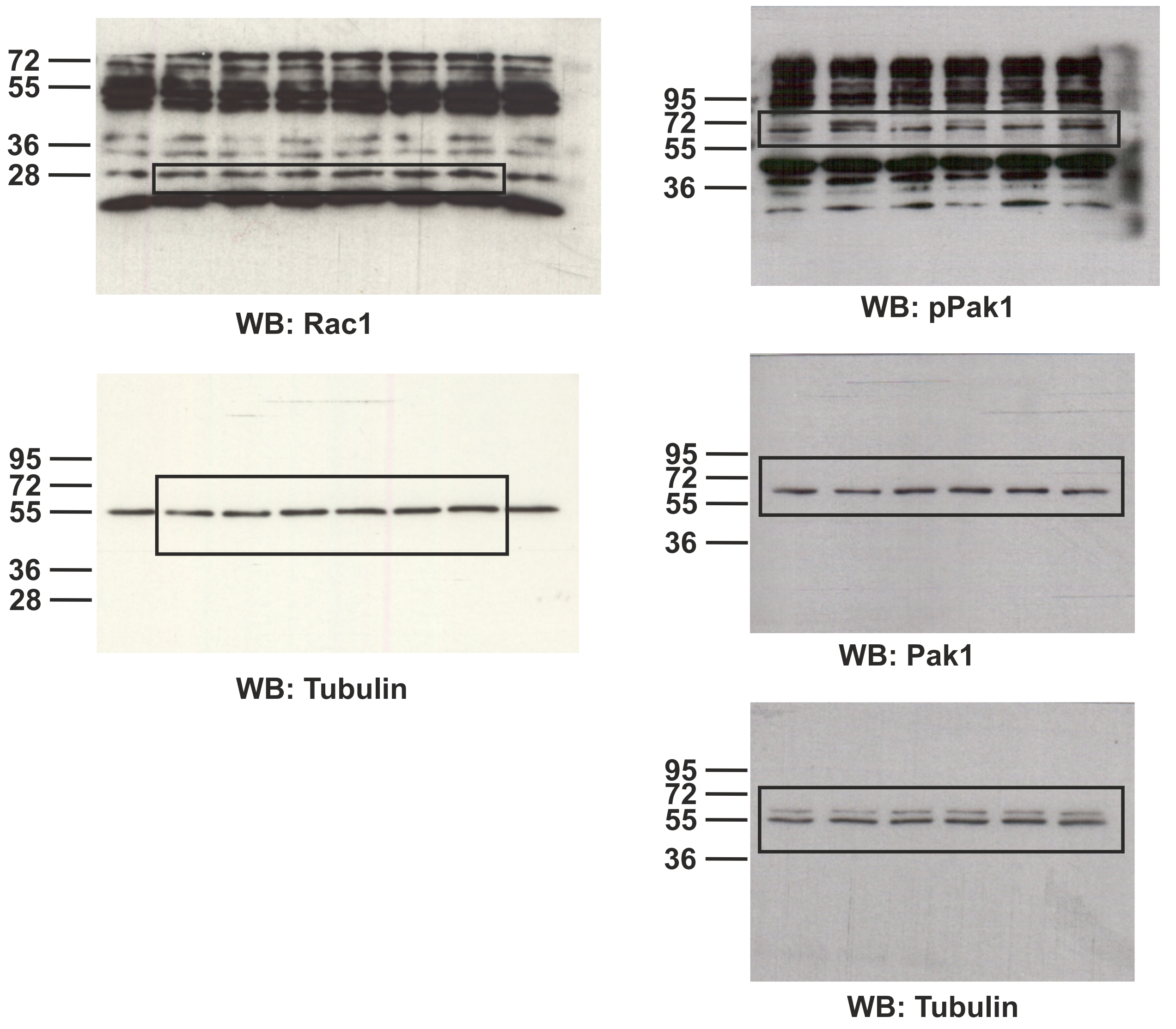

Figure S6. Full scans of the cropped western blots shown in the figures of this study.
Page 4/5, continued in the next page...

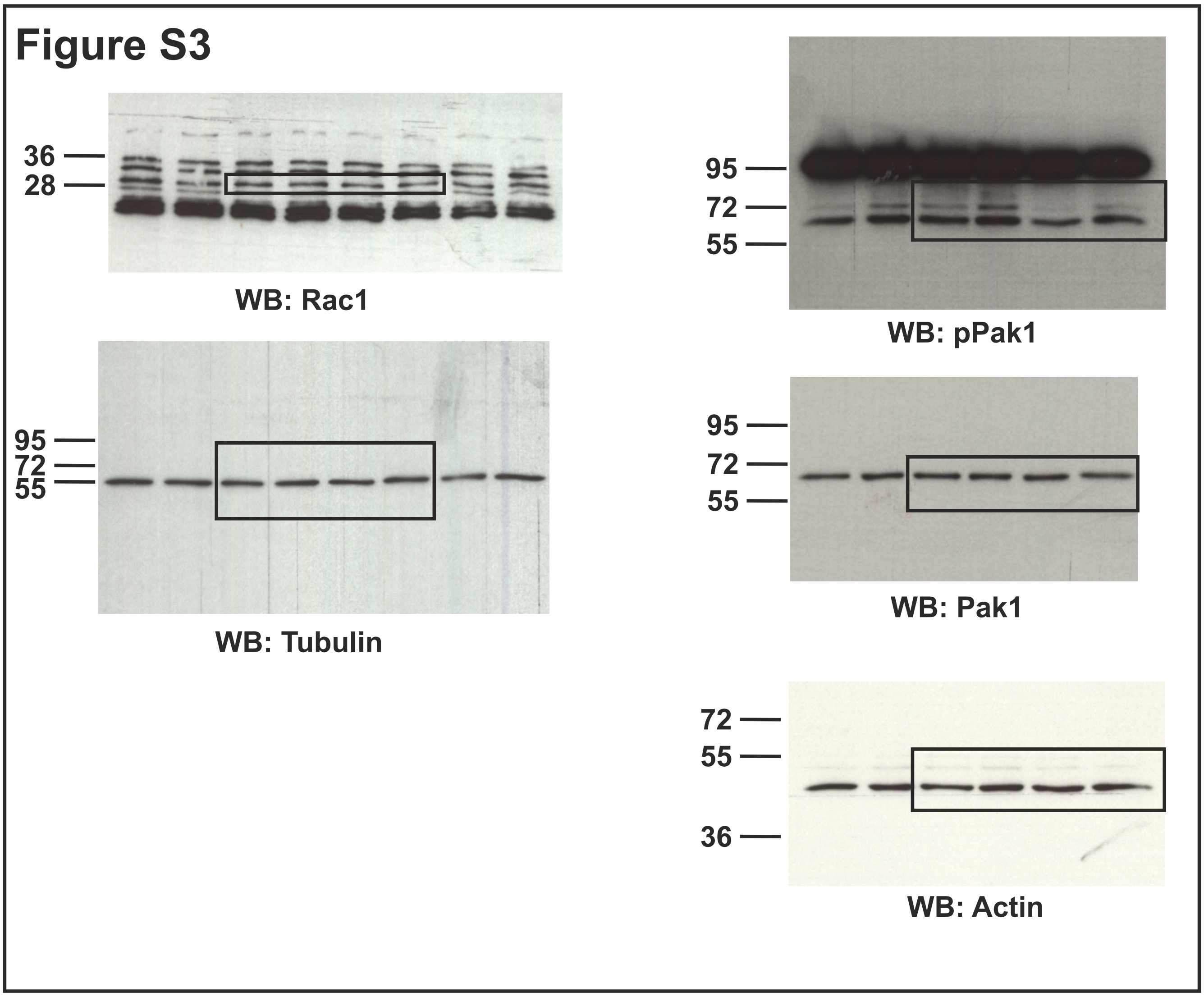

Figure S6. Full scans of the cropped western blots shown in the figures of this study.
Page 5/5.

Table S1. shRNA target sequences used in the study and their respective mRNA depletion efficiencies

Construct	Target sequence(a)	%mRNA(b)	Number of samples
HIF2αKD#1	GGAGCTAACAGGACACAGTAT	93.4±4.6	8
HIF2αKD#2	GGCCAACCAACAGATGGACAA	67±3.9	6
HIF2αKD#3	GTGCATCATGTGCGTGAACTA	60.7±8.1	6

[a] Target sequences were cloned into Retroviral RVH1-puro shRNA vectors as described previously (Schuck et al., 2004).

[b] Indicated as the percent decrease in levels of mRNA (relative to TBP) comparing to control samples.

Table S2. sgRNA target sequences used in the study.

Construct	Target sequence(a)	Number of subclones
HIF2αKO#1	GAAAACATCAGCAAGTTCAT	1
HIF2αKO#2	GAAAGATCATGTCTCCATCT	1
Dock4KO#1	GATCCTGGATCTGCGGCGGC	1
Dock4KO#2	GCACCTGCCGCCGCAGATCC	1

[a] Target sequences were cloned into the vector SpCas9(BB)-2A-GFP (PX458) as described previously (Ran et al., 2013).

Table S3. Verification of gene editing by sequencing of the edited genome region.

Construct	Sequencing result	Editing
HIF2αKO#1 WT allele	GAAAACATCAGCAAGTTCAT	
KO allele 1	GAAAACATCAGCAAG—<br>CATGGGACTCACACAGGTGGAGCTAACAGGACACAGTATCTTTGA	-2bp
KO allele 2	GAAAACATCAGCAAG—<br>CATGGGACTCACACAGGTGGAGCTAACAGGACACAGTATCTTTGA	-2bp
HIF2αKO#2 WT allele	GAAAGATCATGTCTCCATCT	
KO allele 1	A--TGGAGACATGATCTTTCTGTCAGAAAACATCAGCAAGTTCAT<br>GGGACTCACACAGGTGATGCCCTCCTCTGGCTCTTTCAAAAGGGG<br>AAAATGTATCCATTTAGGGGTAGAAATGAGTAG	-2bp
KO allele 2	A--TGGAGACATGATCTTTCTGTCAGAAAACATCAGCAAGTTCAT<br>GGGACTCACACAGGTGATGCCCTCCTCTGGCTCTTTCAAAAGGGG<br>AAAATGTATCCATTTAGGGGTAGAAATGAGTAG	-2bp
Dock4KO#1 WT allele	GATCCTGGATCTGCGGCGGC	
KO allele 1	GATCCTGGATCTGCGGCTGGCAGGTGCTGGTGGGCCACCTGACCCACGACCGG<br>ATGAAGGACGTGAAACGCCACATCACGGCCCGCCTGGACTGGGGCAATGA	+1bp
KO allele 2	GATCCTGGATCTGCGGCCGGCAGGTGCTGGTGGGCCACCTGACCCACGACCGG<br>ATGAAGGACGTGAAACGCCACATCACGGCCCGCCTGGACTGGGGCAATGA	+1bp
Dock4KO#2 WT allele	GCACCTGCCGCCGCAGATCC	
KO allele 1	GGAATCTGCGGCGGCAGGTGCTGGTGGGCCACCTGACCCACGACCGGATG<br>AAGGACGTGAAACGCCACATCACGGCCCGCCTGGACTGGGGCAATGA	+1bp
KO allele 2	GGAGTCTGCGGCGGCAGGTGCTGGTGGGCCACCTGACCCACGACCGGATG<br>AAGGACGTGAAACGCCACATCACGGCCCGCCTGGACTGGGGCAATGA	+1bp

Deletion is marked by -, insertion is marked in yellow, stop codon is marked in grey.

Table S4. Oligonucleotides used for PCR and RT-qPCR.

canis HIF2α F	gagtctgaagctgaggccaa
canis HIF2α R	ccgctctgtggacatgtctt
canis VEGFA F	gtgcattggagccttgcctt
canis VEGFA R	tgtactcgatctcgtcaggg
canis ADM F	cttcgagtgtccagcagcta
canis ADM R	cggtagcgtttgactcggat
canis LOX F	ctccgacgacaacccctatt
canis LOX R	gtggacgcctggatgtagta
canis PAI1F	ggagagacttgtcggcagat
canis PAI1 R	gccgtgaaccagcttcagat
canis SNAI1 F	gatgcacatccgaagccaca
canis SNAI1 R	gggaacaggtcttgcactga
canis SNAI2 F	gggcgcccttaaaatgcaca
canis SNAI2 R	ctgcagatgagccctcagat
canis ZEB1 F	ccaggtggcttacacgtact
canis ZEB1 R	gggtggtgtagaatcagagtca
canis ZEB2 F	aagaaaactggaggaacgcga
canis ZEB2 R	ctcgggggcttctgggtaaa
canis CDH1 F	ccaggcagtcttccaaggat
canis CDH1 R	cacgctgatgactcctgtgt
canis VIM F	gcgtgatgtacgccagcaat
canis VIM R	cacctgtctccggtactcat
canis DOCK4 F	ctgcccgtcgtgttacatca
canis DOCK4 R	ggctggccactatcacatct
